# Chronic cerebral hypoperfusion induces UCP2 expression by decreasing mitochondrial respiratory activities in aging rat brain

**DOI:** 10.1186/1750-1326-7-S1-O6

**Published:** 2012-02-07

**Authors:** Minna Gao, Li Liu, Linhui Wang, Gang Yu, Yu Li

**Affiliations:** 1 Department of Pathology, Yongchuan Hospital, Chongqing Medical University, China; 2 Institute of Neuroscience, Chongqing Medical University, China; 3 Chongqing Key Laboratory of Neurobiology, China; 4 Department of neurology, the first affiliated hospital, Chongqing Medical University, Chongqing 400016, China

## Background

Chronic cerebral hypoperfusion and mitochondria dysfunction have been associated with various neurological and psychiatric diseases, including Alzheimer’s disease and vascular dementia. UCP2 (The uncoupling protein 2) is one member of UCPs, which are a family of mitochondrial anion-carrier proteins. In this study, we intended to investigate the changes of UCP2 and mitochondrial respiratory activities in aging rat brains during chronic hypoperfusion of rats.

## Methods

Chronic cerebral hypoperfusion was induced by ligating of the bilateral common carotid arteries (2VO). Fifty aging male Sprague-Dawley rats aged 12 months and weighing 460~530g, were randomly divided into five groups: a sham-operated group, 1 week, 2 weeks, 3 weeks and 4 weeks after 2VO, with 10 rats in each group. The expressions of UCP2 protein in hippocampus were detected by immunohistochemistry. Mitochondrial cytochrome c oxidase activity was determined with a commercial enzyme assay kit.

## Results

Blood flow was immediately reduced 1 day after 2VO (P<0.001), and did not return to the baseline level even after 28 days. UCP2 protein expression were significantly increased, and reached a peak at 21 days in the hippocampus and in cortex (P<0.01). Mitochondrial cytochrome c oxidase activity was decreased in hypoperfused groups, and had the lowest expression at 21 days after 2VO in hippocampus and cortex (P<0.001).

## Conclusion

Our findings suggested that UCP2 might have important biological roles in protecting against hypoperfusion by decreasing mitochondrial respiratory activities. Further studies are needed to firmly establish this protective effect.

